# Advancing Ground-Based Radar Processing for Bridge Infrastructure Monitoring

**DOI:** 10.3390/s21062172

**Published:** 2021-03-20

**Authors:** Chris Michel, Sina Keller

**Affiliations:** Institute of Photogrammetry and Remote Sensing, Karlsruhe Institute of Technology, 76131 Karlsruhe, Germany; sina.keller@kit.edu

**Keywords:** ground-based interferometric radar, remote measurement, structural health monitoring (SHM), three-dimensional displacement, multiple radar sensors

## Abstract

In this study, we further develop the processing of ground-based interferometric radar measurements for the application of bridge monitoring. Applying ground-based radar in such complex setups or long measurement durations requires advanced processing steps to receive accurate measurements. These steps involve removing external influences from the measurement and evaluating the measurement uncertainty during processing. External influences include disturbances caused by objects moving through the signal, static clutter from additional scatterers, and changes in atmospheric properties. After removing these influences, the line-of-sight displacement vectors, measured by multiple ground-based radars, are decomposed into three-dimensional displacement components. The advanced processing steps are applied exemplarily on measurements with two sensors at a prestressed concrete bridge near Coburg (Germany). The external influences are successfully removed, and two components of the three-dimensional displacement vector are determined. A measurement uncertainty of less than 0.1 mm is achieved for the discussed application.

## 1. Introduction

Critical transport infrastructures like bridges require a periodic assessment of their condition to ensure safe operation. One part of the assessment is monitoring the bridges’ behaviour over time, since changes in this behaviour can indicate damages or deteriorations in the building structure. Depending on the type of load, the monitoring differs with respect to the observed bridge properties. The monitoring of static loads focuses on the displacement of the bridge caused by a known weight. With dynamic loads, the induced weight is generally unknown, since the measurements occur under the regular operation of the bridge. The observed properties are usually displacement or frequency parameters of the decaying vibration after a load on the bridge deck. These parameters are highly dependent on external influences, such as the induced force or the structure’s temperature [1]. Thus, a large number of measurements in different load and weather situations have to be made to receive a reliable estimate of the properties of the bridge.

Traditionally, strain gauges, accelerometers, and other directly contacting sensors are used to monitor the dynamic properties. Since these sensors are mounted directly on the bridge underside, their application can be complicated or even impossible, depending on the bridge’s construction. The installation can also interfere with the regular bridge operation and have a significant setup time (e.g., [2]). In recent years, remote sensing techniques such as ground-based interferometric radar (GBR) have become more common for infrastructure monitoring. GBR is able to measure the displacements of several points with precision in the sub-millimetre range at a sampling frequency of up to 200 Hz [3]. Contactless measurement is achieved by using naturally reflective features at the bridge underside, such as corners or edges, for the reflection of the GBR signal. This measurement type reduces setup time, and the regular operation of the bridge is not disturbed.

### 1.1. Related Work to Infrastructure Monitoring with GBR

GBR can operate in real or synthetic aperture mode. Real aperture mode enables the previously mentioned dynamic displacement monitoring of several points in a structure. It is successfully applied to the monitoring of different infrastructures such as wind turbines [4], buildings [5], towers [6], and bridges [7]. Synthetic aperture mode, by contrast, has a very low sampling frequency, since the sensor head moves along a linear rail or rotates in the horizontal plane. This movement enables an additional azimuth resolution of the observed scene and is used for landslide monitoring [8,9,10], static load tests of bridges [11], or monitoring of large structures such as dams [12,13] or walls [14]. Besides GBR, other remote sensing techniques are applied in the context of bridge monitoring. For example, terrestrial laser-scanning monitors static [15] and dynamic displacements [16]. It offers high spatial resolution but has lower precision compared to GBR. Alternatively, total stations achieve higher accuracy than terrestrial laser scanning but are limited to pointwise measurement [17].

Remote sensing techniques generally require yhr consideration of external and sensor-specific influences on the measurement. One typical external influence to GBR is the change in atmospheric properties. Several approaches for removal of the introduced atmospheric phase are already used for terrain [18,19,20] or dam monitoring [13]. They can also be applied in bridge monitoring [21]. Unwanted reflections from static clutter can cause further influence on the measurement. The resulting systematic deviation can be estimated and removed [20,22]. Since the GBR always measures displacements in the line of sight (LOS), a projection to a Cartesian coordinate system has to be applied. The components of the displacement vector can be determined with multiple simultaneous measurements from different positions, as discussed by Li et al. [23]. Monti-Guarnieri et al. [24] apply the principle in a laboratory setting with two GBRs. Instead of deploying more than one sensor, Miccinesi and Pieraccini [25] use a monostatic/bistatic approach to monitor the three-dimensional displacement vector of a bridge. In a different approach, two components of the displacement vector are determined with a multiple input, multiple output (MIMO) GBR [26]. The principle is also implemented in the synthetic aperture mode [27].

GBR’s most significant advantage over other remote sensing techniques is its high precision. The precision is evaluated in laboratory environments [3,28]. Additionally, comparisons to other sensors such as accelerometers [15], terrestrial laser scanners [29], and displacement transducers [30] are performed.

### 1.2. Objective and Contributions

Our study is motivated by the processing of GBR displacement measurements, especially in the context of complex measurement setups. Complex setups can entail, for example, the use of multiple GBRs or longer measurement durations over several hours. These setups require advanced processing steps that are not usually applied during conventional GBR processing. They are, however, necessary to receive comparable results to the traditionally used sensors. We expand the conventional processing in three main ways:

- Removal of external influences such as disturbances to the signal, static clutter, and changes in atmospheric properties;
- Projection of the displacement vectors measured in LOS by multiple GBR to a Cartesian coordinate system;
- Assessment of the measurement uncertainty.

At first, we discuss the conventional GBR processing, as it is already applied for different monitoring tasks. We then expand this processing, with detection and removal algorithms for external influences to the GBR signal. There are three types of influence to consider. At first, disturbances to the displacement measurements caused by objects passing through the signal are detected and removed. Afterwards, a systematic influence from unwanted reflections is estimated. We modify the principle described in Rödelsperger et al. [20] or Coppi et al. [22] to achieve a more robust estimation, which is especially relevant for long measurement durations over several hours. The third type of external influence is changes in the atmospheric properties, which can severely affect the displacement measurements. To remove these effects, we discuss two approaches, known from the context of ground-based interferometric synthetic aperture radar [18,20], which are applied during the measurement. We also propose a third approach that handles the removal in a post-processing step.

As the GBR always measures displacements in LOS, a projection to a Cartesian coordinate system is needed. The projection is expanded from the one-dimensional approach in the conventional GBR processing to a generalised three-dimensional approach with multiple GBRs [23].

Usually, assessments of GBR’s accuracy are only given in the context of laboratory setups or as comparisons with other sensors. Therefore, we aim to improve the estimation of measurement uncertainty by considering the influence from the applied processing steps. In particular, we consider the uncertainty propagation from the three-dimensional projection. Finally, an application of the GBR processing is shown at a measurement campaign of a pre-stressed concrete bridge.

## 2. Methodology

### 2.1. Fundamentals of GBR

**Figure 1:**
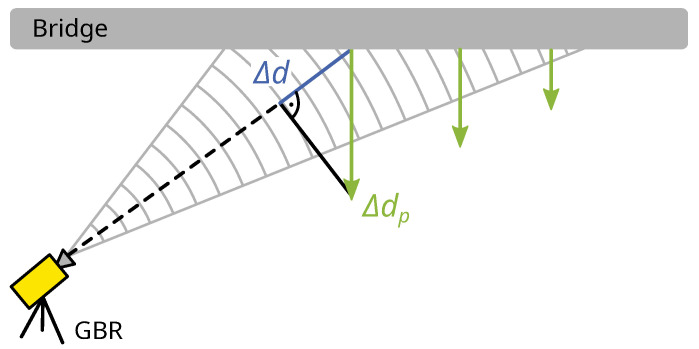
Horizontal view of the measurement principle of GBRs at a bridge underside with LOS-displacement Δd and projected displacement $\Delta d_{p}$.

Commercially available GBRs, like IBIS-FS (IDS, Pisa, Italy), are frequency-modulated continuous-wave radars in the K_u_-band. By modulating the frequency, the GBR is able to discriminate individual targets by their range. The bandwidth of the frequency modulation determines the minimum distance between two targets, called the range resolution. IBIS-FS achieves a range resolution of 0.75 m with a bandwidth of 200 MHz [3]. The displacement of a target within a range-resolution cell is determined by measuring the phase differences in the received signal between consecutive timepoints. In the context of bridge monitoring, the GBR is angled at a bridge underside, as shown in Figure 1, which enables the simultaneous measurement of multiple targets. The targets can only be discriminated by their range, since the GBR has no cross-range resolution.

**Figure 2:**
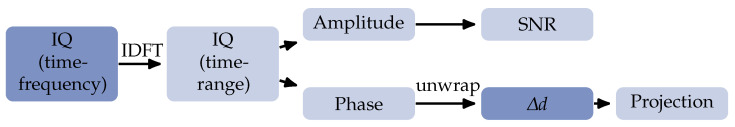
GBR processing of complex raw measurements to projected displacements.

Processing of the raw measurements is needed to receive comparable displacement measurements between different range cells and measurement setups. The GBR measures the in-phase and quadrature components (IQ) of the received signal in the time-frequency domain (see Figure 2). With an Inverse Discrete Fourier Transform (IDFT), these components are transformed to the time-range domain. The amplitude and wrapped interferometric phase are calculated by the absolute and the argument of the transformed IQ components. The wrapped phase has values in the interval [−π,π], leading to jumps when the interval is exceeded. Unwrapping the phase removes these jumps by adding or subtracting 2π when the difference between consecutive samples is greater than π. The displacement Δd in the LOS of the GBR

(1) $$\begin{array}{c} \Delta d=\frac{\lambda }{4\pi }\;\cdot \;\varphi_{uw} \end{array}$$

results from the unwrapped phase $\varphi_{uw}$ and the GBR wavelength λ. The displacement measured in LOS is usually projected to the vertical axis in bridge monitoring. The projected displacement $\Delta d_{p}$

(2) $$\begin{array}{c} \Delta d_{p}=\frac{R}{h}\;\cdot \;\Delta d \end{array}$$

depends on the vertical distance *h* of the GBR to the bridge and the distance *R* to the target. Finally, we can estimate the measurement uncertainty from the signal to noise ratio (SNR) of the amplitude. The SNR results from the amplitude variation [22]

(3) $$\begin{array}{c} \mathrm{SNR}=\frac{m_{a}^{2}}{2\sigma_{a}^{2}} \end{array}$$

where $m_{a}$ is the mean and $\sigma_{a}$ is the standard deviation of the amplitude. An estimate of the displacement uncertainty is then achieved by [20]

(4) $$\begin{array}{c} \sigma_{\Delta d}=\frac{\lambda }{4\pi }\;\cdot \;\frac{1}{\sqrt{2\;\mathrm{SNR}}} \end{array}$$

The described conventional GBR processing is sufficient for most monitoring situations, where the measurement duration is short, and the setup of the instrument is uncomplicated.

However, longer monitoring durations exacerbate the effects of external influences on the GBR signal. Additionally, complex measurement setups, as described in Section 3.1, introduce further external influences, which require advanced processing steps for the detection and removal of these effects.

### 2.2. Overview of Advanced Processing Steps

**Figure 3:**
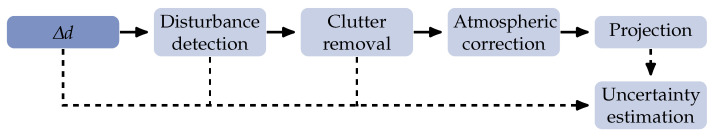
Advanced GBR processing of LOS-displacements.

As shown in Figure 3, the proposed advanced processing steps extend the conventional GBR processing in several ways. Starting with the LOS displacements, the first step is detecting and removing disturbances caused by objects moving through the GBR signal. The removal of the two main types of disturbance, phase jumps and broadband noise, is discussed in Section 2.3. The next step removes clutter from static targets in the same range cells as the monitored bridge targets. The underlying principle of estimating the static clutter is explained, for example, by Rödelsperger et al. [20] or Coppi et al. [22]. We propose a modified circle fit algorithm in Section 2.4 to mitigate the influence of long measurement durations over several hours on the estimation.

With long durations, the influences of changes in the atmospheric properties should also be considered. To correct these influences, two approaches are discussed in the context of ground-based InSAR [18]. The approaches rely on determining the atmospheric influences with additional sensors or a stable reference target. Section 2.5 discusses their implementation for GBR and an alternative post-processing approach.

Finally, the processed LOS-displacements are projected to a Cartesian coordinate system with a generalised approach, proposed in Li et al. [23] and Monti-Guarnieri et al. [24]. With the application of additional GBRs, the components of the three-dimensional displacement vector can be separated. The projection and its influence on measurement uncertainty depending on the GBR setup is discussed in Section 2.6.

### 2.3. Removal of Disturbances

Most of the following GBR processing steps are based on the assumption that the measurements only contain undisturbed bridge displacements. Any disturbances in the GBR signal have to be removed beforehand, in order to not influence the subsequent processing steps. The most common cause of a disturbance is a person, vehicle, or other object passing through the signal at the same range as the target. The GBR cannot distinguish this passing object from the actual measurement target if they are in the same range cell, since the GBR has no cross-range resolution. The passing object is usually moving quickly and is non-deterministic, which leads to broadband noise being superimposed on the phase and amplitude time series. Additionally, a jump in the unwrapped phase time series can occur. Removal of the disturbances changes the variance of the amplitude time series. Therefore the SNR and measurement uncertainty have to be estimated again after the removal.

#### 2.3.1. Phase Jumps

In the detection and removal steps, we separate the disturbances with phase jumps from the disturbances without jumps. Phase jumps are usually caused by a phase unwrapping error. As mentioned in Section 2.1, the unwrapping process removes jumps caused by exceeding the phase interval. A value of 2π is added or subtracted if the phase difference between consecutive samples is greater than π. A disturbance can also cause a difference greater than π without exceeding the phase interval. This is then falsely corrected with 2π during unwrapping, resulting in a jump. The jumps are detected by looking for rapid changes in the unwrapped phase, applying a median filter window, and differencing the resulting values once. Jumps are easily identified by a threshold calculated as a multiple of the standard deviation of the differenced median values. It is important to choose a suitable window length for the median filter. The window length should not be greater than the time between two jumps, as this could mask the individual jumps. Simultaneously, a shorter window can introduce too much noise to the median values. False positives occur if the window length approaches the duration of a vehicle passage.

#### 2.3.2. Broadband Noise

Disturbances without phase jumps can generally be characterised as broadband noise. Typically, the frequency range for displacements of bridges or other large structures is very low. Therefore, a high-pass filter with a cut-off frequency of around 15 Hz eliminates the mostly deterministic components in the low-frequency range. Thus, only sensor noise and the broadband disturbances remain. As the sensor noise can usually be characterised as white noise and is smaller than the noise caused by the disturbances, a threshold is used to detect the disturbances. Ideally, the threshold would be determined as a multiple of the sensor noise standard deviation. However, since the calculation of the standard deviation includes the disturbances, the threshold value is likely too high and false negatives occur. Therefore, the removal of the disturbances is implemented as an iterative process. Every iteration recalculates the standard deviation, thus reducing the threshold until it converges. The final threshold value determines the smallest detectable disturbance for a given standard deviation.

### 2.4. Clutter Removal

**Figure 4:**
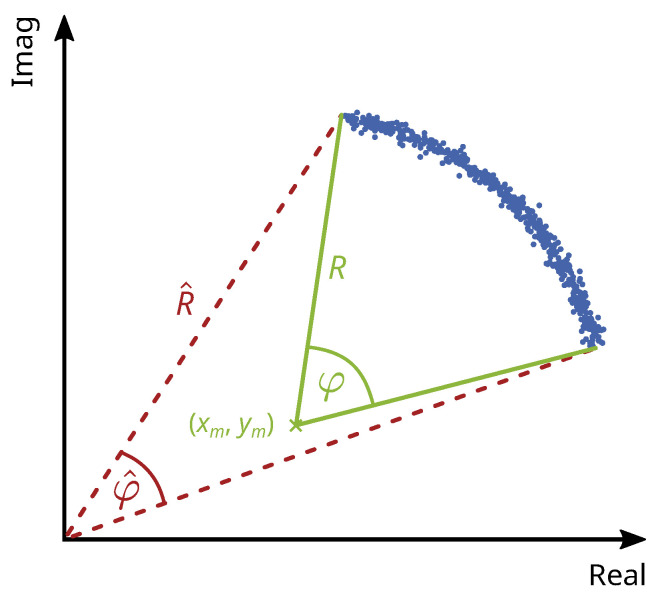
Phasor plot of the circular arc of a moving target with radius *R* and central angle φ superimposed with a static target. The static target adds a systematic deviation to the origin $\left(x_{m},y_{m}\right)$ of the circular arc.

The measurement can be influenced by unwanted targets in the same range cell as the measurement target, since the GBR always measures the sum of the complex IQ components. This clutter can be removed if it results from static targets that have a systematic influence on the measurement [20,22]. The effect is best shown in a phasor plot of the complex signal (see Figure 4). A moving target’s phasor plot shows a circular arc with a radius *R* corresponding to the signal amplitude and a central angle φ corresponding to the signal phase. Static clutter increases the signal amplitude but has no effect on the phase. Therefore it adds a systematic deviation to the origin of the arc, and the phase and amplitude of the moving target are over- or underestimated with $\hat{\varphi }$ and $\hat{R}$. Since the measurement uncertainty is dependent on the mean value of the amplitude, it is over- or underestimated as well. In the following Section 2.4.1, we propose a combination of two circle fit algorithms already used in various other disciplines to estimate the true origin $\left(x_{m},y_{m}\right)$ of the arc. The combination of two algorithms is necessary because bridge displacements usually do not produce a full circle, only a circular arc with a small central angle. The proposed combined approach estimates the origin more reliably. Section 2.4.2 addresses further challenges in circle estimation. For example, we introduce a weight matrix to compensate for an uneven distribution of datapoints, resulting in a more reliable estimation.

#### 2.4.1. Circle Fit Algorithms

Most of the commonly applied circle fitting algorithms use the least squares approaches to determine the circle parameters. The non-linear circle equation

(5) $$\begin{array}{c} F\left(L,X\right)={\left(x-x_{m}\right)}^{2}+{\left(y-y_{m}\right)}^{2}-R^{2}=0 \end{array}$$

is a function of the measured values L=(x,y) and the model parameters $X=(x_{m},y_{m},R)$ of the circle. Least squares minimises the error distances between the measured values and the parameters. The approaches differ with respect to the type of error distances that are minimised. In an algebraic fit, the error distances are derived from an implicit equation [31]. As this equation is linear, the fit is easy to compute but only delivers an approximation of the circle parameters. Alternatively, a geometric fit defines the error distances as the orthogonal distances from the measured values to the model [32]. The circle parameters are updated iteratively with a linearisation of the non-linear equations. Since the linearisation depends on approximate values for the first iteration, we combine both approaches.

First the algebraic fit determines approximate values for the circle parameters. We introduce a substitution in Equation (5)

(6) $$\begin{array}{cc} x^{2}+y^{2} & =2\;x\;x_{m}+2\;y\;y_{m}-x_{m}^{2}-y_{m}^{2}+R^{2} \end{array}$$

(7) $$\begin{array}{cc} z & =a\;\cdot \;x+b\;\cdot \;y+c \end{array}$$

In this substitution, the measured values x and y are treated as constants, so that the function is linear and only depends on the substitute parameters *a*, *b*, and *c*, which are estimated in linear least squares. The origin and radius of the circle result from a resubstitution of these parameters.

**Figure 5:**
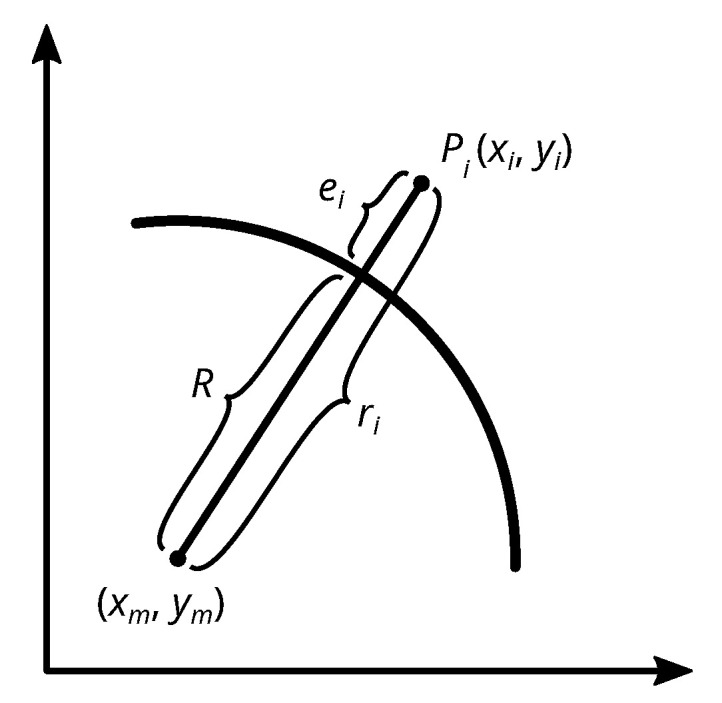
The orthogonal distances $e_{i}$ are defined as the residuals between the radii $r_{i}$ of the points $P_{i}$ and the circle radius *R*.

In a second step, the geometric fit uses these approximate values to iteratively calculate the final circle parameters. The orthogonal distances are defined as the residuals e between the radii r of the measured values and the circle radius *R* (see Figure 5):

(8) $$\begin{array}{c} e=R-r \end{array}$$

As proposed in Niemeier [33], the radii of the points

(9) $$\begin{array}{c} r=\sqrt{{\left(x-x_{m}\right)}^{2}+{\left(y-y_{m}\right)}^{2}} \end{array}$$

are treated as measured values in Equation (8). This means that the function to be minimised is only dependent on the parameters. As the function is non-linear, the equations have to be linearised at the approximate values $X^{0}=\left(x_{m}^{0},y_{m}^{0},r^{0}\right)$ of the parameters

(10) $$\begin{array}{cc} {\left.\frac{\partial e}{\partial x_{m}}\right|}_{X^{0}} & =\frac{x-x_{m}^{0}}{r^{0}} \end{array}$$

(11) $$\begin{array}{cc} {\left.\frac{\partial e}{\partial y_{m}}\right|}_{X^{0}} & =\frac{y-y_{m}^{0}}{r^{0}} \end{array}$$

(12) $$\begin{array}{cc} {\left.\frac{\partial e}{\partial R}\right|}_{X^{0}} & =1 \end{array}$$

The measured values are reduced by the approximate circle radius $R^{0}$

(13) $$\begin{array}{c} l=r-R^{0} \end{array}$$

With the Jacobi-matrix A containing the partial derivatives from Equations (10) to (12), the least squares is set up

(14) $$\begin{array}{c} l+e=A\;\cdot \;\Delta X \end{array}$$

The solution ΔX is updated iteratively with the approximate parameters $X^{0}$, until it converges.

#### 2.4.2. Challenges in Circle Estimation

The estimation of a circle or circular arc depends on several factors. Most importantly, it is influenced by the central angle of the arc. The approximate radius from the algebraic fit is underestimated with a decreasing angle, which usually leads to the geometric fit converging to a local minimum. Consequently, the estimated origin deviates from the true origin, which induces a systematic deviation in the resulting displacement measurement.

Another important influence is disturbances that are not detected by the proposed algorithms in Section 2.3. The broadband noise superimposed on the complex radar signal acts as outliers to the circle estimation, resulting in a poor fit. Remaining disturbances or other kinds of outliers can be handled in an outlier detection or with a robust modification of the circle estimation.

The distribution of the datapoints within the circular arc can also have an influence on the estimation. Generally, most datapoints are concentrated around the (arbitrarily defined) zero phase, especially for low frequencies of vehicle passages. This leads to a poor fit at the outer points of the arc, because the circle radius is over- or underestimated. We can reduce this effect by introducing a weight matrix P in Equation (14) of the geometric fit

(15) $$\begin{array}{c} l+e=A\;\cdot \;P\;\cdot \;\Delta X \end{array}$$

The diagonal of the weight matrix contains the phase value for every datapoint so that the outer points are weighted higher than the points near zero phase.

### 2.5. Atmospheric Influence

Changes in the atmospheric refractive index can have severe effects on microwave measurements. The microwave propagation delay leads to an additive term in the interferometric phase measurement [18]. This additive term is the atmospheric phase

(16) $$\begin{array}{c} \varphi_{\mathrm{atmo}}=\frac{4\pi }{\lambda }\;\cdot \;R\Delta n \end{array}$$

which is dependent on the distance *R* to the target and the change in the refractive index Δn. A correction of the atmospheric phase is especially important for longer measurement durations and can be achieved with different methods. One approach is to calculate the refractive index with empiric formulas and measured atmospheric properties; most importantly, humidity and temperature [34]. For this, the atmospheric properties need to be mostly homogenous on the whole measurement path, which can only be reasonably assumed for distances under a couple of hundred metres.

A second approach uses a stable measurement target for the correction of the atmospheric phase. If the displacement of this target is zero, the measured phase can be wholly attributed to changes in the atmospheric properties (and sensor noise) [20]. The phase of this stable target then reduces the other target’s phase. To keep the influence of noise and the inhomogeneity of the atmosphere low, the stable target should have an SNR that is equal to or higher than the other targets, and should be in close proximity to them.

We propose a third approach, which relies on post-processing of the displacement measurements. This can be applied if certain conditions are met: the traffic intensity is low, so that, in a given time frame, the duration of vehicle passages is shorter than the duration of no passages. Additionally, the duration of a vehicle passage should only be a few seconds. Assuming the atmospheric properties are only changing slowly, the atmospheric phase can be removed by subtracting a moving median of the displacement measurements. With a window length of 15 s to 60 s, the long-wave atmospheric phase is removed without influencing the measurements of the vehicle passages.

### 2.6. Three-Dimensional Projection

Since the GBR always measures displacements in LOS, a projection to a Cartesian coordinate system is needed to generate comparable results between different measurement setups. Additionally, the measurements in LOS may contain displacement components from more than one bridge axis. In general, bridges have the highest displacement amplitude in the vertical axis. Displacements in the direction of the transversal or longitudinal axis are smaller, but can still influence the measurement in the vertical axis depending on the setup [11]. By using more than one GBR simultaneously, it is possible to separate the displacement components for different axes.

#### 2.6.1. Principle

**Figure 6:**
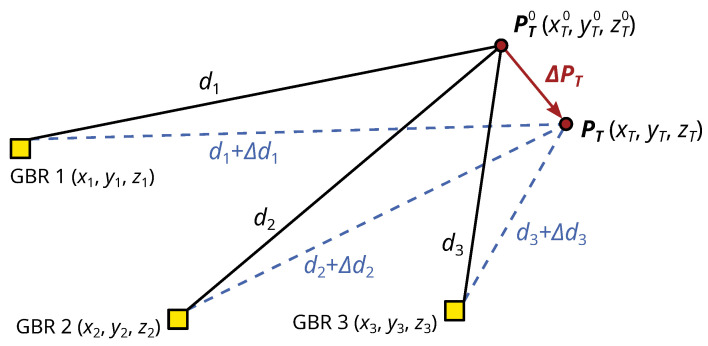
Schema of GBR and target geometry for the transformation of LOS-displacements Δd to displacements in a Cartesian coordinate system Δp.

The projection uses the principle described by Li et al. [23] and Monti-Guarnieri et al. [24], who define a transformation of displacements measured in LOS to a three-dimensional displacement vector in a Cartesian coordinate system. Figure 6 shows the geometry of the measurement setup with the GBR coordinates $P_{i}=\left[x_{i},y_{i},z_{i}\right]$ and the initial target coordinates $P_{T}^{0}=\left[x_{T}^{0},y_{T}^{0},z_{T}^{0}\right]$. The target is displaced by the three-dimensional vector $\Delta P_{T}=\left[\Delta x_{T},\Delta y_{T},\Delta z_{T}\right]$ to the new coordinates $P_{T}=\left[x_{T},y_{T},z_{T}\right]$. This displacement is measured indirectly by the GBRs with the LOS-displacements Δd. To derive a projection from Δd to $\Delta P_{T}$, we define the distance

(17) $$\begin{array}{c} d_{i}=\sqrt{{\left(x_{i}-x_{T}^{0}\right)}^{2}+{\left(y_{i}-y_{T}^{0}\right)}^{2}+{\left(z_{i}-z_{T}^{0}\right)}^{2}} \end{array}$$

between the i-th GBR (i=1⋯3) and the target. Since the target displacements are differential, we can linearise the distances $d=\left[d_{1},d_{2},d_{3}\right]$ at the initial target coordinates $P_{T}^{0}$

(18) $$\begin{array}{cc} d(P_{T}) & \approx d\left(P_{T}^{0}\right)+\frac{\partial d}{\partial P_{T}}{|}_{P_{T}=P_{T}^{0}}\;\cdot \;\left(P_{T}-P_{T}^{0}\right) \end{array}$$

(19) $$\begin{array}{cc} d\left(P_{T}\right)-d\left(P_{T}^{0}\right) & =\frac{\partial d}{\partial P_{T}}{|}_{P_{T}=P_{T}^{0}}\;\cdot \;\Delta P_{T} \end{array}$$

The difference between the distances are the LOS-displacements Δd. With the Jacobi-matrix A composed by the partial derivatives, we can write the projection as

(20) $$\begin{array}{c} \Delta d=A\;\cdot \;\Delta P_{T} \end{array}$$

The target displacements in the Cartesian coordinate system result from inverting the Jacobi-matrix

(21) $$\begin{array}{c} \Delta P_{T}=A^{-1}\;\cdot \;\Delta d \end{array}$$

When using only two GBR, we have to make the assumption that the displacement in one coordinate axis is zero. The corresponding line and column to this axis is then excluded from the Jacobi-matrix, and the inversion is possible.

#### 2.6.2. Variance Propagation

There are two components to consider when evaluating the influence of the projection on measurement uncertainty. One component is the propagation of the uncertainty depending on the GBR and target geometry; the other component is the uncertainty of the geometry measurement itself. First, the measurement uncertainty σ of all GBRs

(22) $$\begin{array}{cc} \Sigma_{\Delta d} & =\sigma_{\Delta d}\;\cdot \;I \end{array}$$

is propagated with the Jacobi-matrix A, resulting in the projected measurement uncertainty

(23) $$\begin{array}{c} \Sigma_{\Delta p}=A^{-1}\;\cdot \;\Sigma_{\Delta d}\;\cdot \;{A^{-1}}^{T} \end{array}$$

To account for the uncertainty in the geometry measurement, we propose a Monte Carlo simulation, which models the uncertainties in GBR and target coordinates and shows their influence on the projection. Generally this influence is small compared to the influence of the propagation, but it can become significant for certain setups where the target cannot be clearly identified or localised.

These two considerations enable an a priori review of different measurement setups regarding their influence on measurement uncertainty. Some examples are discussed in the following section.

#### 2.6.3. Scenarios

**Figure 7:**
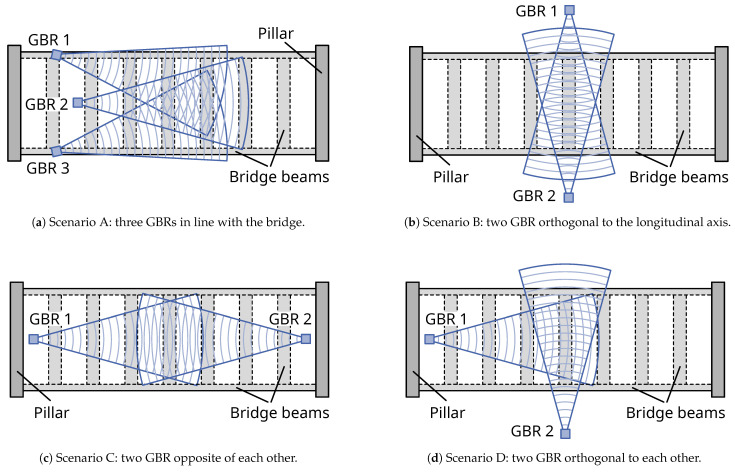
Vertical view of possible measurement setups at a bridge with two longitudinal and consecutive orthogonal beams.

The placement of the GBRs mainly depends on the bridge and the respective measurement situation, but it can also be optimised for different objectives. In this section, we discuss possible scenarios for the measurement setup and their advantages and disadvantages. Figure 7 shows four scenarios for a common bridge type. The bridge has two longitudinal beams spanning from one pillar to the other, as well as consecutive orthogonal beams.

Scenario A uses three GBR to determine all components of the three-dimensional displacement vector. The GBRs are placed near one bridge pillar, facing the orthogonal beams from roughly the same direction, thus maximising the number of measurement targets equal to all GBRs. There has to be some variation between the positions in at least two coordinate axes to determine all three displacement components. This scenario is useful for a monostatic/bistatic configuration, as described by Miccinesi and Pieraccini [25], or in the case of inaccessible areas under one bridge side. However, due to the proximity of the GBRs to each other, the projection has a large influence on measurement uncertainty. Additionally, an orthogonal beam may be entirely contained within one range cell. Torsional movements could, therefore, not be determined.

Low measurement uncertainty can be achieved with Scenario B, which places two GBRs directly opposite each other. With this setup, only the vertical and longitudinal displacement components can be determined. It is also not possible to identify torsional movements.

In Scenario C, two GBRs are placed orthogonal to the bridge, enabling the determination of the vertical and transversal displacement components. The influence of the projection on measurement uncertainty is also lower, as in Scenario A. However, this setup significantly decreases the number of measurement targets, since only the two longitudinal beams reflect the GBR signal.

Scenario D is a compromise specific to our measurement situation described in Section 3. Since our GBRs operate on the same signal frequency, interference can only be avoided if they are placed orthogonal to each other. The setup results in the measurements from GBR 1 being composed of both the vertical and longitudinal displacement components. For the centre of the bridge, the longitudinal component can be assumed to be negligible. This assumption may not be the case further to the pillars [11].

## 3. Results

To evaluate the advanced processing steps described in the previous sections, we performed several measurements at a bridge in Schneckenlohe, Germany. The results for the entire processing workflow of these measurements are shown in the following sections.

### 3.1. Measurement Setup

**Figure 8:**
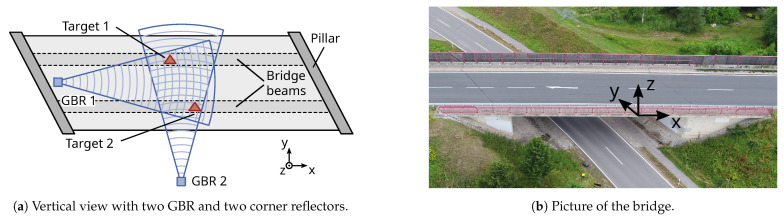
Measurement setup at Schneckenlohe (Germany) on 22 October 2019.

The pre-stressed concrete bridge is part of the federal road B303 and crosses the district road KC29 in Schneckenlohe, Germany (see Figure 8b). It is 26 m long and has two parallel concrete beams in the longitudinal direction (*x*-axis). Good signal reflection from the beams can be achieved when the GBR is placed directly orthogonal to them. Consequently, the measurements in LOS are composed of the vertical (*z*-axis), but also the transversal (*y*-axis) displacement of the bridge. To separate the displacement components, we use an additional GBR placed under the bridge, in line with the beams. Figure 8a shows a similar measurement setup to Scenario D from Section 2.6.3. This is a necessary compromise to avoid interference between the GBRs. To ensure sufficient signal reflection, we install two corner reflectors at the centre of each beam. The reflectors do not occupy the same range cell because of the trapezoidal bridge shape. Determining the torsional movement is, therefore, still possible. Since the two GBRs are independent sensors, their time information needs to be synchronised. Synchronisation is achieved by cross-correlating the displacement time series. In the following sections, we discuss the processing of a 3.5 h long measurement, starting at 12:30 h on 22 October 2019.

### 3.2. Phase Jumps

**Figure 9:**
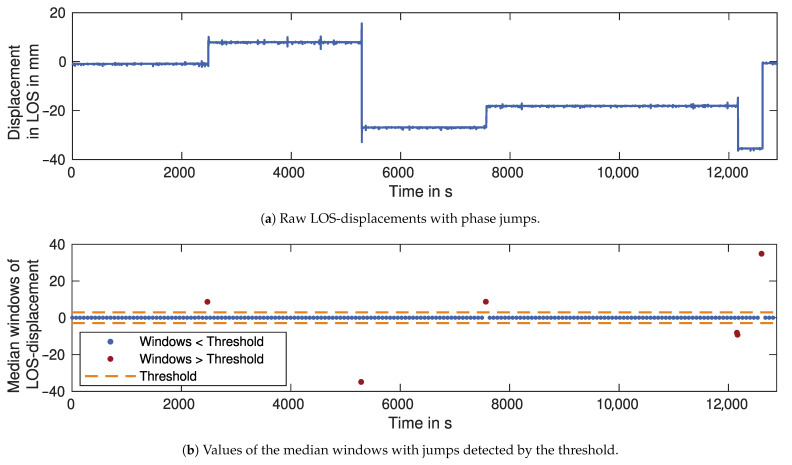
Detection of phase jumps in raw LOS-displacements for Target 1 measured by GBR 1.

The signal paths of both GBRs cross the district road, where passing vehicles cause disturbances in the signal. The disturbances are mostly broadband noise, but they can also contain phase jumps when the phase is incorrectly unwrapped. Figure 9a shows the LOS-displacements of Target 1, measured by GBR 1. The clearly visible jumps cause shifts in the mean values in the order of several millimetres. For detecting these jumps, median windows with a window size of 10 s are calculated and differenced once, as described in Section 2.3.1. The resulting values are displayed, together with a threshold determined as a multiple of their standard deviation in Figure 9b. All jumps are correctly detected by this threshold and removed from the displacement time series.

### 3.3. Broadband Noise

**Figure 10:**
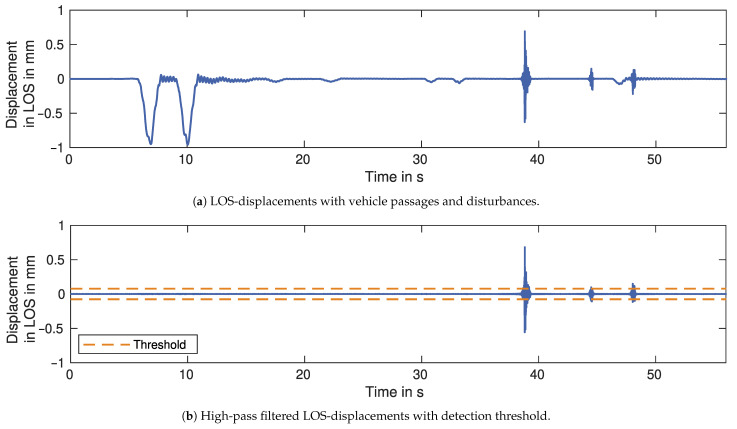
Section of LOS-displacements for Target 1 measured by GBR 1.

As discussed in Section 2.3.2, vehicles or other objects passing through the signal path cause broadband noise to be superimposed on the displacement time series. The detection and removal of these disturbances are shown in Figure 10a for a section of the LOS-displacements of Target 1. The time series contains displacements of the bridge caused by trucks and cars passing over it, as well as disturbances in the form of broadband noise. Since the bridge displacements mostly contain low-frequency components which are smaller than 10 Hz, the disturbances are easily detected by high-pass filtering of the time series and the application of a threshold calculated as a multiple of the standard deviation of the filtered time series. Figure 10b shows the LOS-displacements after applying the high-pass filter with a cut-off frequency of 15 Hz. All disturbances exceed the threshold and, therefore, are correctly detected.

For a more comprehensive evaluation, we assess the proposed algorithm’s detection accuracy for the entire measurement setup. The disturbances are manually labelled as ground truth to determine recall and precision. The results for the two GBRs and two targets are shown in Table 1. About 240 disturbances were detected in the 3.5 h long measurement. This number is expected to be roughly the same for all four measurements, since the signal paths of both GBRs cross the district road KC29, which is the primary source of the disturbances. Generally, almost all disturbances are correctly detected, which is indicated by the high recall values above 99%. A small number of additional disturbances are detected that are not present in the displacement measurement. This leads to precision values ranging from 94% to 98%.

### 3.4. Clutter Removal

**Figure 11:**
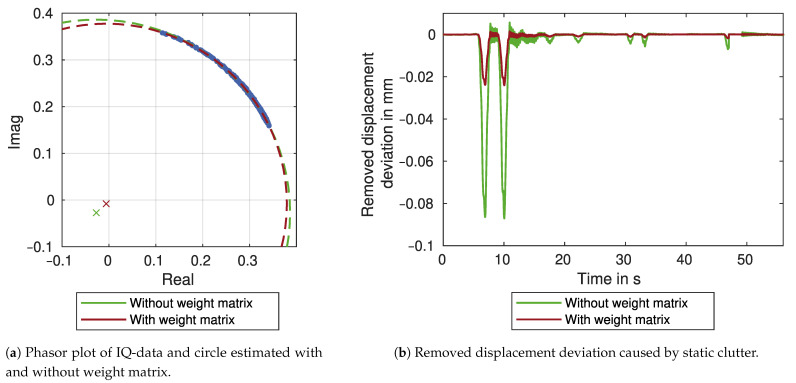
Clutter removal for a section of the LOS-displacements for Target 1 measured by GBR 1.

Moving objects and measurement targets at the bridge cannot be distinguished if they are in the same range cell. The corresponding parts of the measurement have to be removed, as discussed in the previous section. However, static targets have a systematic influence on the measurement, which can be determined by the circle fit algorithm proposed in Section 2.4. Figure 11a shows the estimation results in a phasor plot of the complex IQ components. The circle parameters are estimated without a weight matrix (green) and with a weight matrix (red), as described in Section 2.4.2. For both results, the circle centres differ slightly from the coordinate system’s origin. Therefore, static clutter is present in the signal. By subtracting the deviation from the complex signal and reprocessing it, the static clutter is removed. Figure 11b shows the deviation that is removed from the section of the displacement time series. It is proportional to the LOS-displacements with values in the order of 8% for the estimation without a weight matrix (green) and 2% for the estimation with a weight matrix (red). See also Figure 10a as a comparison.

### 3.5. Atmospheric Correction

**Figure 12:**
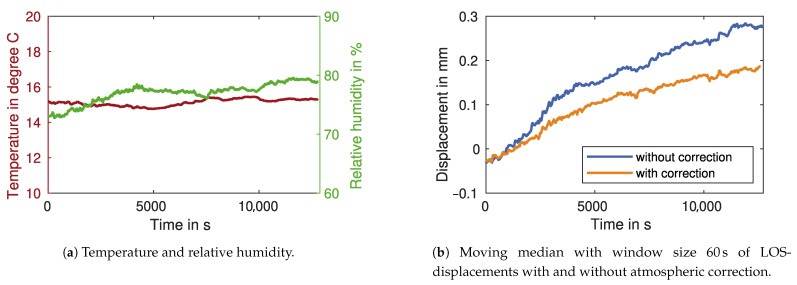
Atmospheric correction with measured atmospheric parameters.

For the atmospheric correction, we install atmospheric sensors near GBR 1. The sensors measure temperature and relative humidity, as shown in Figure 12a. During the measurement, the humidity rises by about 7%. The temperature varies only minimally in the order of 1 ${}^{\circ }C$. Figure 12b shows the LOS-displacements with and without the atmospheric correction (see Section 2.5). A moving median is applied to the graphs, to better visually compare the drift in both time series. The approximately linear drift is only partially reduced by the atmospheric correction. To remove the remaining drift, the third approach discussed in Section 2.5 can be used.

### 3.6. Two-Dimensional Projection

Since the GBR usually faces the bridge underside at an angle, the LOS-displacements are not comparable between different bridge targets or if the measurement setup is changed. Therefore, a projection to a Cartesian coordinate system has to be applied. For most bridges and our studied bridge in Schneckenlohe, we expect the highest displacement amplitude in the vertical direction (*z*-axis, see Figure 8). The applied measurement setup is also able to determine the displacement component orthogonal to the driving direction (*y*-axis). The displacement in the longitudinal axis parallel to the driving direction (*x*-axis) is assumed to be zero, as the measurement targets are in the middle of the bridge. This allows us to use only two GBRs with the previously discussed measurement setup.

**Figure 13:**
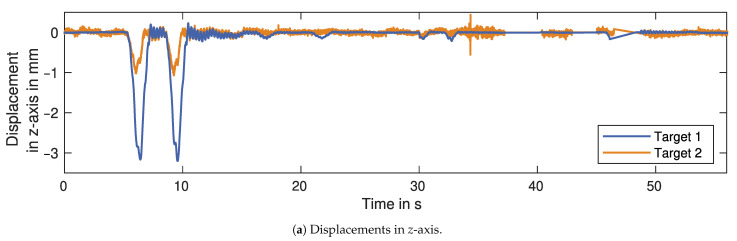

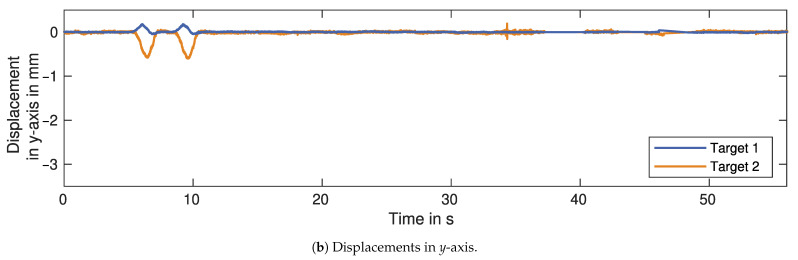
Section of projected displacements for both targets.

Figure 13 shows the projection results for a section of the LOS-displacements. The vertical displacement is displayed for both targets in Figure 13a. For the two truck passages at 7 s and 10 s the vertical displacement is higher for Target 1, which indicates that the trucks are passing over the bridge on the north lane (right-hand traffic). The transversal displacement shown in Figure 13b is much smaller than the vertical displacement. Additionally, the truck passages generates positive values for Target 1 and negative values for Target 2. This means the bridge beams are displaced outwards from the centre, away from each other. Besides the displacements caused by the trucks, the cars passing over the bridge only generate small vertical displacements and no significant transversal displacements.

### 3.7. Evaluation of Measurement Uncertainty

Every processing step has an influence on the measurement uncertainty, either through changes in the amplitude time series or through a propagation. The measurement uncertainty is initially estimated from the SNR of the amplitude, as discussed in Equations (3) and (4). Table 2 shows these estimates for the displacement in LOS in a range of 0.02 mm to 0.14 mm. Removing the disturbances reduces the amplitude variance, which results in lower estimates for the measurement uncertainty. The removal of static clutter influences the mean value of the amplitude, thus increasing or lowering the uncertainty. After clutter removal, the uncertainty values are in the range of 0.02 mm to 0.03 mm, except for Target 1 measured by GBR 1, which has a significantly lower value. These values are now propagated, as discussed in Section 2.6.2. The resulting uncertainty for the projected displacement in the *y*- and the *z*-axis is lower than 0.1 mm in all cases.

Additionally, the uncertainties of the coordinate measurements are considered. By using a total station, the coordinates of the GBRs zero point can be determined very accurately. However, the target coordinates are much more inaccurate because there is usually not one exact reflective point, even when using a corner reflector. Therefore, we estimate the measurement uncertainty after the projection with a Monte Carlo simulation. The simulation models three normally distributed inputs: the measurement uncertainties after clutter removal (see Table 2), the GBR coordinate uncertainty estimated with 0.01 m, and the target coordinate uncertainty $\sigma_{coord}$. The inputs are propagated with Equation (21) using the maximum negative LOS-displacements shown in Figure 10a. Initially, $\sigma_{coord}$ is set to zero, confirming the results from the previous variance propagation. We estimate $\sigma_{coord}$ to be 0.05 m for a corner reflector and 0.30 m for a natural reflective target like a bridge beam. Table 3 shows the simulation results. The coordinate uncertainty of a corner reflector does not significantly increase the measurement uncertainty, except for the *z*-axis of Target 1. If, instead, a natural reflective target was used, the measurement uncertainty would rise substantially. The *z*-axis of Target 1 again experiences the most significant increase, to almost ten times the value without a coordinate uncertainty.

## 4. Discussion

In this section, we discuss the results of our proposed processing steps, applied exemplarily at the measurement campaign in Schneckenlohe, and the influence of each step on the subsequent processing. We start with the detection and removal of disturbances from the LOS-displacements.

The main source of disturbances at the bridge in Schneckenlohe is traffic on the district road crossing the GBR signal. Phase jumps, as one type of disturbance, are caused by phase unwrapping errors from large disturbances. These usually originate from large vehicles such as trucks or buses. In our case, the occurrence of these vehicles is rather low, and all phase jumps were correctly detected. For a higher traffic volume, the window size may have to be reduced to detect all jumps. However, smaller window sizes can lead to false positives, since vehicle passages would also be detected as jumps.

The second type of disturbance is broadband noise caused by cars or people crossing the GBR signal. We detect broadband noise with a threshold applied to the high-pass-filtered displacements. The detection accuracy depends on the threshold. A higher threshold improves the precision but worsens the recall. Generally, a high recall value is preferred to a high precision value. Most studies avoid the occurrence of disturbances, either through their measurement setup or with short measurement durations. To further establish GBR as an alternative to the traditionally used sensors, it is, however, crucial to implement the proposed disturbance removal. The subsequent processing steps, such as clutter removal and the estimation of measurement uncertainty, rely on data without disturbances or other types of outliers, which is achieved with our high recall value. It also enables measurements over several hours, which are necessary to reliably estimate the bridge’s dynamic properties [1].

The next processing step removes static clutter by estimating the centre of a circular arc in the phasor plot. This estimation is challenging, especially for long measurement durations. By introducing a weight matrix, the estimation compensates the unequally distributed datapoints. The results differ significantly between the estimation with and without a weight matrix, which demonstrates the significant influence a small deviation in the circle centre has on the LOS-displacements. Therefore, the benefit of removing static clutter has to be weighed against the uncertainty introduced by the circle estimation. By compensating the unequally distributed datapoints with the weight matrix, this uncertainty is significantly reduced, and clutter removal becomes beneficial for GBR processing.

The atmospheric correction with empiric formulas only partially removes the approximately linear drift from the LOS-displacements, for which tehre can be multiple reasons. For example, the atmospheric properties may not have been determined accurately enough, either because of inhomogeneous atmospheric conditions or inaccurate sensors. Effects unrelated to the atmosphere can also cause drift. The tripod of the GBR, for example, can slowly settle in the ground over time, causing a positive drift. Additionally, the expansion of the bridge can also lead to drift. However, since the structure’s temperature usually lags severely behind the air temperature, we cannot confirm this with our measurements. Removing the remaining drift with the post-processing approach can be useful for comparisons to other sensors or further processing. The approach is applicable for most measurement situations where the vehicle passages have a short duration and the atmospheric properties are changing slowly.

The last processing step is the projection of the displacements measured in LOS to a Cartesian coordinate system. Since we use two GBR, we can determine two of the three displacement components. However, the benefit of this approach is not only the determination more than one component but also the reduction in the influence from the longitudinal or transversal component on the vertical component. Without this approach, the measurements of GBR 2 would be significantly affected by the transversal component. GBR 1 could still be affected by the longitudinal component, although we assume this to be insignificant compared to the other two components. This assumption should be confirmed with an alternate measurement setup, as described with Scenario A or B in Section 2.6.3. The setup could be implemented with a bistatic approach or a MIMO GBR [25,26].

Lastly, the measurement uncertainty is evaluated. Table 2 shows that every processing step has a significant influence on the measurement uncertainty. The disturbance removal always leads to an equal or lower uncertainty, since it only removes parts of the amplitude time series, improving the SNR. Clutter removal can both increase and lower the amplitude’s mean value, and thus also the uncertainty. The result of the projection’s variance propagation highly depends on the positions of the GBRs relative to each other. A consideration of the coordinate uncertainty may not be necessary if the reflective point of a target is precisely defined with a corner reflector. However, this precise definition is usually not possible for most natural reflective features leading to a significant influence on measurement uncertainty. Since these considerations can be performed without real measurement data, it is advisable to analyse the uncertainty prior to a measurement campaign only by means of the GBR positions. Compared to laboratory environments [3,28], the achieved uncertainties are plausible. Nevertheless, they might be higher for other bridges and measurement setups with longer ranges to the targets and without corner reflectors. Generally, this demonstrates the importance of determining the measurement uncertainty, since it highly depends on the applied processing and the measurement setup.

## 5. Conclusions and Outlook

This paper introduces advanced processing steps for GBR measurements in the context of bridge monitoring. Based on the conventional processing approach, we propose advanced steps focusing on three main aspects: the removal of external influences, projection of the displacement vectors measured in LOS to a Cartesian coordinate system, and an assessment of the measurement uncertainty. The proposed steps enable specific measurement setups, which were not previously possible with conventional GBR processing.

For long measurement durations, it is crucial to remove disturbances, static clutter, and atmospheric propagation delay. These external influences not only affect the measurement results, but also the subsequent processing steps. We therefore implement a disturbance detection with high precision and recall values to remove this influence as much as possible. Similarly, a stationary object located in the same range cell as the target causes a systematic deviation. The estimation of this deviation is improved by introducing a weight matrix, which compensates for unequally distributed datapoints. Lastly, the propagation delay from changes in the atmospheric properties is removed with atmospheric sensor data, and an alternative post-processing approach.

In conventional GBR processing, the LOS displacement vector is generally projected to the vertical axis, thus disregarding the influence from the other two displacement components. We therefore expand the one-dimensional projection with a generalised three-dimensional approach to separate the displacement vector components. Finally, we evaluate the measurement uncertainty from the acquired data itself and in the context of the applied processing steps.

In future work, we aim to investigate the accuracy of GBR measurements further. For example, in the clutter removal step, the uncertainty of the estimation itself should also be considered. Furthermore, the results of the three-dimensional projection should be validated with a different sensor type.

## Figures and Tables

**Table 1:** Detection results for the displacement measurements in Schneckenlohe on 22 October 2019.

GBR	Target	Number of Disturbances	Threshold	Precision	Recall
GBR 1	Target 1	239	0.01 mm	94%	99.2%
GBR 1	Target 2	235	0.08 mm	97.8%	99.6%
GBR 2	Target 1	232	0.02 mm	96.5%	99.1%
GBR 2	Target 2	249	0.01 mm	97.6%	100%

**Table 2:** Measurement uncertainty of the displacements for the two targets and GBRs.

Target	GBR	LOS	After Removal of Disturbances	After Clutter Removal	After Projection to *y*-Axis	After Projection to *z*-Axis
Target 1	GBR 1	0.019 mm	0.007 mm	0.002 mm	0.045 mm	0.036 mm
	GBR 2	0.034 mm	0.005 mm	0.033 mm		
Target 2	GBR 1	0.142 mm	0.027 mm	0.027 mm	0.036 mm	0.095 mm
	GBR 2	0.029 mm	0.008 mm	0.019 mm		

**Table 3:** Measurement uncertainty of the projected displacements considering the uncertainty of the target coordinates $\sigma_{coord}$.

Target	$\sigma_{\mathrm{coord}}$	After Projection to *y*-Axis	After Projection to *z*-Axis
Target 1	0.05 m	0.046 mm	0.067 mm
	0.30 m	0.060 mm	0.342 mm
Target 2	0.05 m	0.037 mm	0.096 mm
	0.30 m	0.043 mm	0.124 mm

## Data Availability

The data are not publicly available as they contain sensitive information about the investigated bridge structure.
